# Evolution of a microbial nitrilase gene family: a comparative and environmental genomics study

**DOI:** 10.1186/1471-2148-5-42

**Published:** 2005-08-06

**Authors:** Mircea Podar, Jonathan R Eads, Toby H Richardson

**Affiliations:** 1 Diversa Corporation, 4955 Directors Place, San Diego, CA 92131 USA

## Abstract

**Background:**

Completed genomes and environmental genomic sequences are bringing a significant contribution to understanding the evolution of gene families, microbial metabolism and community eco-physiology. Here, we used comparative genomics and phylogenetic analyses in conjunction with enzymatic data to probe the evolution and functions of a microbial nitrilase gene family. Nitrilases are relatively rare in bacterial genomes, their biological function being unclear.

**Results:**

We examined the genetic neighborhood of the different subfamily genes and discovered conserved gene clusters or operons associated with specific nitrilase clades. The inferred evolutionary transitions that separate nitrilases which belong to different gene clusters correlated with changes in their enzymatic properties. We present evidence that Darwinian adaptation acted during one of those transitions and identified sites in the enzyme that may have been under positive selection.

**Conclusion:**

Changes in the observed biochemical properties of the nitrilases associated with the different gene clusters are consistent with a hypothesis that those enzymes have been recruited to a novel metabolic pathway following gene duplication and neofunctionalization. These results demonstrate the benefits of combining environmental genomic sampling and completed genomes data with evolutionary and biochemical analyses in the study of gene families. They also open new directions for studying the functions of nitrilases and the genes they are associated with.

## Background

Having colonized virtually every environment, bacteria and archaea have evolved enzymatic solutions for a wide range of metabolic biochemical transformations [1,2]. Studying enzymes derived from organisms inhabiting these environments is important for understanding how microbes adapt, react to and transform the environment. The overwhelming majority of microbial species remain however uncultivated [3]. A variety of functional and sequence-based approaches have been developed for discovering and characterizing genes, operons and even entire genomes directly from the environment, collectively referred to as metagenomics or environmental genomics [4]. The use of environmental genomics has already led to important discoveries such as genes responsible for novel biological functions [5], microbial community metabolic traits [6-8] and dramatic increases in the diversity of various enzyme families [9,10]. Subsequent biochemical and evolutionary analyses can strengthen the biological end ecological inferences even before organisms that carry that genetic information are isolated in culture [11-13]. From a practical perspective, microbial environmental genomics has been a successful approach for the discovery of enzymes for a broad spectrum of biotechnological applications [14-17].

To gain insight into the evolution of function in a gene family that has been extensively sampled by environmental genomic screening and characterized biochemically, we focused on bacterial nitrilases. These enzymes are members of the carbon-nitrogen hydrolase superfamily which catalyze the hydrolysis of a wide range of non-peptide carbon-nitrogen bonds [18-20]. The nitrilase family hydrolyzes nitriles to their corresponding carboxylic acids, releasing ammonia. This reaction is likely involved in detoxification of xenobiotics and nitriles produced as defense chemicals by other microorganisms and plants, as well as in secondary metabolite biosynthetic pathways. Nitrilases appear to be rare in bacteria (out of over 150 sequenced bacterial genomes only 10 contain nitrilase genes). Recently, over 130 nitrilases were identified by functional screening of hundreds of environmental DNA libraries, for use in industrial biocatalysis applications [9]. Those enzymes were characterized biochemically and classified into six subfamilies, four of them with no representatives in known bacterial species. It was found that a number of enzymatic properties (substrate specificity and enantioselectivity) were specific to subfamilies and, in some cases, correlated with the biogeography and ecology of the environmental samples.

The role of gene duplication, natural selection and functional diversification in the evolution of the nitrilase gene family is unknown. The correlation of distinct enzymatic properties with the different genes subfamilies suggest that nitrilases have diverged functionally to accommodate distinct biological roles in microbial communities that occupy various ecological niches. Functional divergence is the result of changes in selection pressure and is often accompanied by associations with novel gene clusters or operons which encode for enzymes with coupled metabolic activities. To begin addressing some of these aspects, we analyzed the genetic neighborhoods of all available nitrilase genes, identified conserved patterns of conserved gene clustering relative to biochemical data and phylogeny and propose a hypothesis on nitrilase evolution involving gene duplications and Darwinian selection.

## Results and discussion

### The nitrilases from cultivated bacteria belong to clade-specific gene clusters

**Figure 1:**
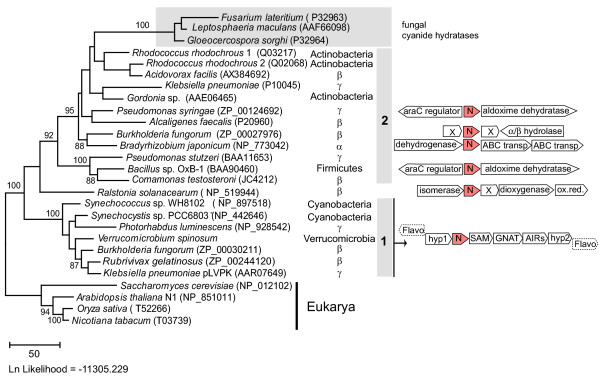
Maximum likelihood tree of nitrilases from known bacterial species (accession numbers are in parentheses). Bootstrap support values are indicated for the major groups only. The schematic organization of the gene clusters that contain a nitrilase ORF is shown for species where that sequence information is available.

Bacterial nitrilases (137 environmental sequences and 10 sequences from cultivated species) have been recently classified into six major clades [9] that we refer to as subfamilies. We analyzed more recently released genome sequences and found an additional nine novel nitrilases. Phylogenetic analysis of a sequence dataset consisting of all nitrilase genes from cultivated bacteria shows that 18 sequences belong to subfamilies one and two (Fig. 1). The level of sequence similarity among these 18 enzymes is quite high, ranging from 50–70% pairwise identity in subfamily one to 30–40% in subfamily two. The relationships between the different nitrilases do not reflect the taxonomy of their host organisms. Additionally, for several genera or species that harbor two nitrilases (*Pseudomonas*, *Klebsiella pneumoniae *and *Burkholderia fungorum*), the genes belong to different subfamilies/clades, suggesting ancient gene duplications or acquisition by horizontal gene transfer (HGT). *Rhodococcus rhodochrous *on the other hand contains two closely related nitrilases, suggesting a more recent gene duplication event. Supporting the possibility of HGT, one of the nitrilase genes we identified by database mining is in the plasmid pLVPK of *Klebsiella pneumoniae*, which may be transferable to other bacteria. Also, several fungal cyanide hydratase genes form a clade deeply nested within subfamily two of bacterial nitrilases, suggesting HGT acquisition from bacteria, followed by neofunctionalization. The paucity of nitrilase genes in bacterial genomes makes it difficult to evaluate the contribution of the different evolutionary events (duplications, gene loss and HGT) to the observed distribution and the functional significance of the presence of different types of enzymes in related organisms.

**Figure 2:**
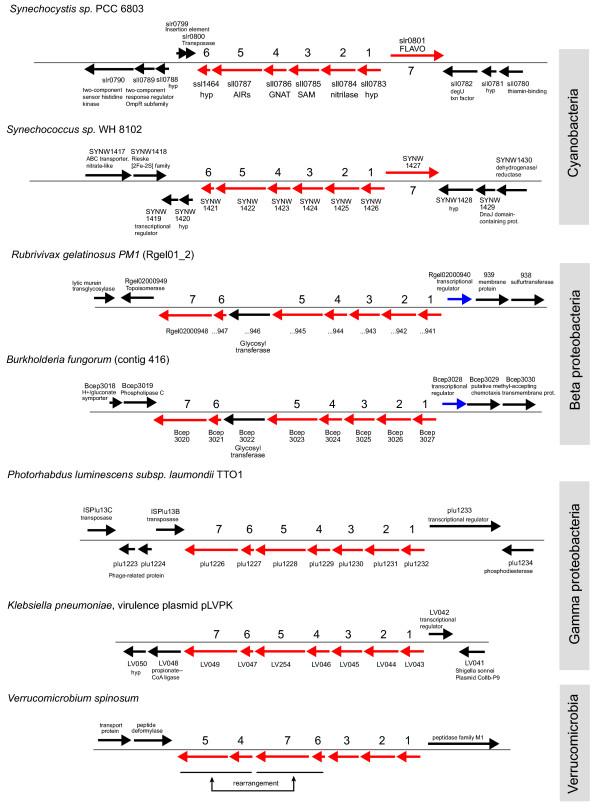
Organization of gene clusters around the subfamily 1 nitrilases in sequenced bacterial genomes. The highly conserved gene cluster Nit1C is flanked by unrelated genomic neighbourhoods in the different species. Gene names are based on the available genomic annotation.

In bacteria, genes are often organized in clusters (e.g. operons, regulons) that reflect involvement in a common metabolic process or association in a supramolecular complex [21-23]. To determine if nitrilase function could be inferred from the nature of the surrounding genes, we analyzed those genes in the available genomic data. We found that all of the known seven subfamily 1 nitrilase genes (six genomic and one on a plasmid) belong to a conserved and previously undescribed cluster of seven genes, Nit1C (Figure 1 and Figure 2). Six of the coding sequences are on the same DNA strand, separated by few or no intergenic nucleotides and are likely part of an operon/regulon. This hypothesis is supported by analysis using a recent method for operon prediction [24] although we could not identify conserved transcription factor binding sites in the upstream region. The genes in this predicted operon occur in the order (1) hypothetical protein, (2) nitrilase, (3) radical S-adenosyl methionine superfamily member, (4) acetyltransferase, (5) AIR synthase, and (6) hypothetical protein. The seventh gene encodes a predicted flavoprotein, putatively involved in K^+ ^transport and is located either at the beginning of the cluster but on the opposite strand (cyanobacteria *Synechocystis sp*. PCC6803 and *Synechococcus sp*. WH8102) or as the last gene of the cluster, in the same orientation as the others (proteobacteria *Burkholderia fungorum*, *Rubrivivax*, *Photorhabdus luminescens *and *Klebsiella pneumoniae*). In *Verrucomicrobium spinosum*, the cluster has been rearranged, as ORFs 6 and 7 occur in between ORFs 3 and 4. Yet another variation exists in the betaproteobacteria *Burkholderia *and *Rubrivivax *where a glycosyltransferase gene is inserted between ORFs 5 and 6. These slight variations in the cluster architecture correlate to the major taxonomic bacterial groups (Cyanobacteria, Beta- and Gamma proteobacteria). Outside of Nit1C there is no conservation between the different species in terms of genes or metabolic functions encoded by gene clusters. The presence of genes associated with mobile DNA elements (transposases, IS elements) immediately downstream of the Nit1C clusters in *Synechocystis *and *Photorhabdus *and the apparent interruption of a large polyketide synthase pathway by the nitrilase cluster in *Photorhabdus *may indicate HGT or internal chromosomal rearrangements.

In the case of subfamily 2, gene neighborhood information was available for only four of the twelve genes from cultivated bacteria. In *Bacillus sp*. and *Pseudomonas syringae*, the nitrilase gene is apparently co-transcribed with a downstream phenylacetaldoxime dehydratase gene and preceded by an araC transcription factor transcribed from the other strand. The other nitrilase genes (from *Burkholderia*, *Bradyrhizobium *and *Ralstonia*) are part of unrelated clusters (Figure 1).

In addition to the nitrilases from completed genomes of cultivated bacteria, we searched for such enzymes in two large environmental sequence datasets: the acid-mine drainage microbial mats [7] and the Sargasso Sea [10] using BLASTP. No nitrilases were found in the acid-mine dataset. In the Sargasso Sea dataset we identified 17 nitrilases that were full-length or long enough to be phylogenetically informative. Three of the genes appear to be eukaryotic while eight bacterial genes are close relatives to nitrilases from *Synechoocccus *or *Burkholderia*. The remaining six genes do not appear to have close relatives among known nitrilases and belong to subfamilies 2, 4 and 5 [see Additional file 1]. Finding so few nitrilase genes in such a large dataset suggests that for uncovering the sequence space of a gene family, functional screening of a large number of samples from very different environments is more efficient than deep sequence coverage of one or a few environments.

### Nitrilases associated with different types of gene clusters have distinct enzymatic properties

For the nitrilase genes identified from environmental DNA, the identity of the host organism is unknown. However, because those libraries were constructed using fragments of genomic DNA several times larger than the average nitrilase gene length (~1 kb), we also analyzed the the gene neighborhood of the environmental nitrilase. Because of the highly conserved nature of the Nit1C cluster and its occurrence in distant taxa of bacteria, we first focused on mapping its distribution among the environmental nitrilase clones. We found that the Nit1C cluster is strictly confined to a group of subfamily 1 nitrilases that includes the seven genes identified in completed genomes and 14 of the environmental ones. Four of the subfamily 1 nitrilases from the Sargasso Sea dataset had small flanking sequences and we identified the presence of the Nit1C type genes (ORFs 1 or 3), similar to those of their close relatives from *Synechococcus *and *Burkholderia*. However, because of their incomplete length, those sequences were not included in further analyses.

**Figure 3:**
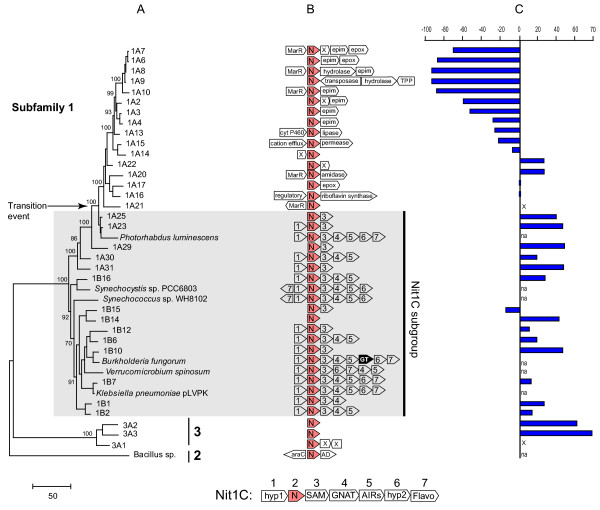
(**A**). Protein maximum likelihood tree of subfamily 1 nitrilases. The tree was arbitrarily rooted with sequences from the two most closely related subfamilies 2 and 3. Numbers at nodes represent bootstrap support (not shown if <50). (**B**). Diagram of the gene clusters that include the nitrilase ORF. For environmental genes, the information was limited by the size of the genomic insert. (**C**). Histogram representing enzymatic enantioselectivity (R or S) on hydroxyglutaronitrile, based on data from [9](na, not assayed; x, not active).

The nitrilase genes that belong to the Nit1C cluster are indicated on a maximum likelihood phylogenetic tree calculated using the subfamily 1 genes as well as several outgroup sequences from subfamilies 2 and 3 (Figure 3A). Since the size of the genomic insert in the environmental clones was limited, not all the Nit1C genes were identified; however, we did not find evidence to suggest that the cluster was different in any of the host genomes (Figure 3B). We also identified a more recent evolutionary event that marks the loss of nitrilase association with the Nit1C cluster. After that transition event (TE), nitrilase genes are no longer associated with a highly conserved gene cluster. Instead, they are flanked by genes encoding MarR transcriptional regulators, epimerases, epoxide hydrolases and other ORFs. These latter genes were not so highly conserved in their order as those found in the Nit1C cluster. No cultivated bacteria that contain nitrilases from this group have been found so far.

The sister group of subfamily 1 nitrilases, subfamily 3, consists of only three environmental type genes. We had sufficient flanking sequence to determine the nature of the neighboring genes for only one of the genes (3A1), flanked by two hypothetical ORFs with no identifiable homologs. Therefore, the Nit1C cluster appears to have originated with and is restricted to a subset of subfamily 1 nitrilases. The more distantly related nitrilases from subfamilies 4, 5 and 6 have no apparent associations with a conserved gene cluster (data not shown).

In our previous study [9] we uncovered a number of correlations between the biochemical properties of the environmental microbial nitrilases and their phylogenetic classification. Distinct gains or losses of activity or switches in enantioselectivity coincided with the evolutionary events that led to the formation of the main subfamilies. One of the most interesting findings was a reversal in enantioselectivity (R to S) that occurred in subfamily 1, against the model substrate hydroxyglutaronitrile. To correlate the differences in types of gene clusters with the nitrilase biochemical properties, we graphed the available hydroxyglutaronitrile activity data on the side of the phylogenetic tree (Figure 3C). With one exception (1B15), the enzymes that belong to the Nit1C group are R-enantioselective on hydroxyglutaronitrile. The transition event (TE) marks changes in biochemical properties leading to enantioselectivity reversal. The first enzyme not associated with Nit1C (1A21) was inactive on that substrate, while the next diverging ones (1A20, 1A22, 1A16, 1A17) were R-selective or not enantioselective (low bootstrap values do not support a robust branching order). However, the next statistically supported clade (1A14 and above in the Figure 3A tree) show a reversal of enantioselectivity followed by a steep increase in selectivity to values over 95%.

### Analysis of the subfamily 1 nitrilase gene clusters

Having determined that subfamily 1 nitrilases belong to two distinct subgroups based on their associated gene clusters and enzymatic properties, we analyzed the nitrilase neighboring genes for clues to their individual metabolic roles. First in the Nit1C cluster, ORF1 proteins are highly conserved in length (160–163 amino acids) and sequence (>60% identity between any two genes). However, no other homologs were found using standard searching techniques of current databases. Using HMM structural homology modeling (Superfamily 1.63 server) [25], we tentatively assigned the hypothetical protein 1 to the YchN1-like superfamily and fold, whose biochemical activity is unknown. Next in the cluster is the nitrilase gene. The third gene encodes a member of the radical SAM superfamily (Pfam 04055), enzymes that catalyze a wide variety of radical-based reactions through reductive cleavage of S-adenosylmethionine at an iron-sulfur center [26]. The Nit1C SAM genes form a strongly supported clade (~50% average sequence identity), most closely related to bacterial and archaeal genes annotated as biotin synthase-related enzymes (COG2516) [see Additional file 2]. ORF4 in the Nit1C cluster also forms a clade of closely related sequences and belong to the GCN5-related N-acetyltransferase (GNAT) superfamily (Pfam 00583) [27]. These enzymes are involved in antibiotic detoxification as well as in histone acetylation in eukaryotes. The closest homologs to the Nit1C GNAT genes are a number of other acetylases from bacteria like *Rhodobacter *and *Enterococcus *[see Additional file 2]. The fifth gene in the cluster encodes members of the large 5'-phosphorybosyl-5-aminoimidazole synthase-related proteins superfamily (AIRS, Pfam 00586). Enzymes in this superfamily are involved in de novo purine biosynthesis, selenophosphate synthesis, or maturation of NifE hydrogenase. These genes form a unique clade, most closely related to a group of archaeal genes encoding phosphoribosylformylglycinamide synthases [see Additional file 2]. The last invariant position in the cluster, ORF6, encodes a protein of approximately 100 amino acids. While the sequence identity between the individual genes surpasses 70%, we could not find any other relatives to these genes by any sequence analysis approach. The seventh ORF of Nit1C is located at either end of the cluster, on either coding strand. This gene is a member of the pyridine nucleotide-disulphide oxidoreductases (Pfam 00070, COG2072), that include flavin-containing monooxygenases and flavoproteins involved in K^+ ^transport. The closest relatives to the Nit1C genes are putative monooxygenases found in several species of *Pseudomonas *[see Additional file 2]. All Nit1C genes form clusters of closely related sequences within their respective superfamilies, suggesting a common function, possibly in a pathway for detoxification of plant or microbial defense compounds.

Members of the nitrilase clade that split after the transition event are exclusively of environmental origin, with no sequence representatives in characterized bacterial species. Approximately two thirds of the nitrilases in this group are associated with genes encoding a MarR transcriptional regulator, epimerases and epoxide hydrolases. MarR genes (PFam 01047) are transcriptional repressors controlling the expression of the Mar operon, involved in multiple antibiotic resistances [28]. The nitrilase-associated MarR genes form a specific clade, most closely related to genes from *Xanthomonas *and *Desulfitobacterium *(30–40% identity) [see Additional file 3] and are always upstream of the nitrilase gene. The location of the epimerase and epoxide hydrolase varies somewhat, the epimerase ORF being usually between the nitrilase and the epoxide hydrolase ORFs. Epimerases are a large class of enzymes that reversibly determine stereochemical inversions of hydroxyl substituents in carbohydrates, participating in numerous metabolic pathways [29,30]. The nitrilase-associated epimerases form a unique clade in which the relationship between the genes parallels that of their associated nitrilases. Their closest relatives are epimerases from species of *Streptomyces *(~35% identity) [see Additional file 3]. Epoxide hydrolases belong to the large superfamily of alpha-beta fold hydrolases and hydrate chemically reactive epoxides to more stable dihydrodiols. This reaction is of major importance in detoxification of a large number of endogenous epoxide metabolites and xenobiotic compounds in all organisms [31]. The association of all these genes with nitrilases could indicate the requirement for coupled reactions under the transcriptional control of MarR, perhaps involved in detoxifying sugar-based cyanogenic compounds in soils rich in decaying plant material.

### Positive selection as a possible driving force for nitrilase functional diversification

The observed changes in associated gene clusters and in enzymatic properties suggest that the hypothetical gene duplication in subfamily 1 was followed by nitrilase recruitment to novel metabolic functions, possibly under selective constraints. A powerful approach to studying changes in the selective pressure in protein encoding genes involves calculation of the nonsynonymous/synonymous substitution rate ratio (ω = dN/dS) (reviewed in [32,33]). A ratio below one indicates negative (purifying) selection, restricting amino acid changes that could interfere with a well-established protein function, while ω = 1 suggests that the gene evolves neutrally. On the other hand, a ratio significantly higher than one may indicate a selective advantage for fixation of amino acid changes. This can be considered evidence of positive selection associated with functional divergence after events such as gene duplications or changes in the environment (e.g. [34,35]).

Using a relative rate test [36], we first investigated the rate variation between the branches flanking the transition event (1A23/1A25 and 1A21). A likelihood ratio test based on a three-taxon tree (consisting of 1A25 and 1A21 as test sequences and 1A29 as outgroup) compared the null hypothesis (equal rates for both branches following the transition event) with an alternative model with unconstrained rates. The null model was rejected (P = 2 × 10^-6^, df = 1), supporting a 5.6 times faster overall rate for the 1A21 lineage than for 1A25, which has maintained the Nit1C association. A rate increase is predicted when gene duplication is followed by functional divergence and could occur because of positive Darwinian selection or an increase in fixation of neutral mutations as result of relaxation of functional constraints [37-40].

To test if positive selection acted along the nitrilase lineages flanking the cluster transition event, we used a maximum likelihood (ML) approach based on codon substitution models [34]. These models take into account sequence features such as transition-transversion rate biases, codon usage variation and allow testing hypotheses at specific branches in a phylogeny by employing heterogeneous ω values among sites and lineages. Positive selection can also be investigated using a parsimony-based method, there being some controversy on to which of the two methods is more reliable [41-43].

The tree used for ω estimation was obtained based on the nitrilase DNA sequences, focusing on the genes around the transition event (Figure 6A). The first set of likelihood models that we used, site-specific [44], assume variations in the selective pressure across sites but no variations among individual genes. Using these models we determined that purifying selection has a dominant role across subfamily 1 nitrilases (ω = 0.04) (Table 1). This is reflected in the large number of conserved amino acids: 86 invariant (~25% of sites) and 149 conserved at 90% level in this data set. No significant positive selection signal was identified using this category of models. However, since these models average the substitution ratios of individual sites over all lineages, they are known to lack sensitivity in detecting positive selection that acts only along a few lineages (e.g. [44,45].

**Table 1:** Parameter estimates, likelihood scores and identified selected sites under various models. Branch numbers refer to Figure 4A. Parameters indicating positive selection are in bold. A likelihood ratio test (LRT) is used to compare a pair of nested models: one which accounts for sites with ω > 1 and one which does not (the null model). To accept or reject the ω > 1 hypothesis, twice the log-likelihood difference in the scores is compared with a χ^2 ^distribution with the degrees of freedom equal to the difference in the numbers of parameters between the two models. When ML detects lineages with ω > 1, an empirical Bayes analysis identifies sites under positive selection and calculate posterior probabilities that provide a measure of confidence for that prediction.

**Model**	**p**	**l**	**Parameter estimates**	**Positively selected sites**	**Likelihood Ratio Test**
M0:one ratio	1	-11903.5	ω = 0.0418	none	

Site-specific models					
M1:neutral (K = 2)	1	-13195.5	p_0 _= 0.298, p_1 _= 0.702	not allowed	
M3:discrete (K = 2)	3	-11627.6	p_0 _= 0.6, p_1 _= 0.4, ω_0 _= 0.012, ω_1 _= 0.098	none	

Branch-site models					
Branch 1					
Model A	3	-13160.0	p_0 _= 0.3, p_1 _= 0.70, p_2_+p_3 _= 0, ω_2 _= 0	none	
Model B	5	-11627.6	p_0 _= 0.4, p_1 _= 0.6, p_2_+p_3 _= 0 ω_0 _= 0.098, ω_1 _= 0.012, ω_2 _= 0	none	
					
Branch 2					
Model A	3	-13188.7	p_0 _= 0.296, p_1 _= 0.688, **p_2_+p_3 _= 0.016, ω_2 _= 129.6**	Q157 (P = 0.77), Q203 (P = 0.999), T41, Q157, Y184, N200, Q203, R284 (P > 0.9)	LRT vs. M1 2Δl = 6.8, P = 0.03, df = 2
Model B	5	-11621.4	p_0 _= 0.356, p_1 _= 0.59, **p_2_+p_3 _= 0.05** ω_0 _= 0.1, ω_1 _= 0.0125, **ω_2 _= 9.7**		LRT vs. M3 (K = 2) 2Δl = 6.2, P = 0.04, df = 2

**Figure 4:**
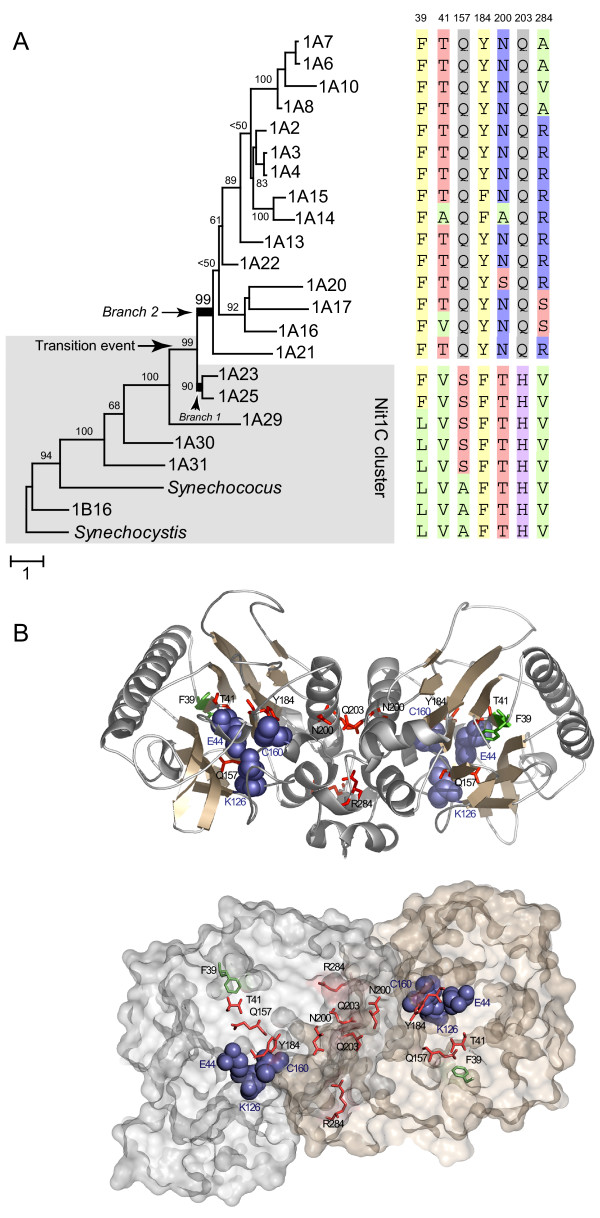
(**A**) Maximum likelihood tree for subfamily 1 nitrilases used to test for positive selection. Branch lengths are scaled to the mean number of substitutions per codon site under model M3. Branches 1 and 2 indicate lineages tested for positive selection signal, following the transition event. The sequences illustrate the variability across the clade at positions identified under positive selection. (**B**). A three dimensional model of the 1A21 nitrilase dimer. Shown are the catalytic triad (blue) and the residues under positive selection (red). Residue 39, invariant before and after the transition event, is shown in green.

To investigate if adaptive evolution acted alongside branches around the transition event, we also used a more recently developed set of maximum likelihood models, which allow the ω ratio to vary among both sites and lineages [46]. These models are more sensitive in detecting positively selected sites along a pre-specified lineage of interest ("foreground" branch) as compared to the rest of the genes ("background" branches). These models were applied to the two lineages that followed the transition event (branches 1 and 2 in Figure 4A). For branch 1, which belongs to the Nit1C nitrilases and served as a negative control, we did not detect any positive selection signal. Branch 2 represents the basal lineage for the group of nitrilase genes that have lost the Nit1C cluster association, potentially having led to nitrilase neofunctionalization. A significant positive selection pressure (ω = 9.7 under model B) was detected for that lineage, the empirical Bayes analysis pointing to residues T41, Q157, Y184, N200, Q203 and R284 as being the selection target. These amino acid positions may represent hot spots for changes in substrate specificity or other nitrilase enzymatic properties. The variation of those aminoacids across the subfamily is shown in Figure 4. Shown also is a site (residue 39) that is invariant before the transition event then changes with that event and becomes again invariant.

High resolution structures are not yet available for nitrilases. However, the structures of two homologs, the *C. elegans *NitFhit protein and the *Agrobacterium radiobacter *N-carbamoyl-D-amino acid amidohydrolase (D-NCAase) have been solved [47,48]. Both proteins form tetramers with two dimer subunits and revealed a novel four layer α-β-β-α fold. It is believed that all members of the nitrilase superfamily share this fold and the catalytic triad Glu-Lys-Cys in the active site. A three dimensional model of 1A21 (the first nitrilase outside the Nit1C group) was derived based on the D-NCAase structure coordinates, and used to map the location of the residues under positive selection at the CTE. Three of those, T41, Q157 and Y184, were found to be buried within the protein, close to the catalytic triad (E44, K126, C160) (Figure 4B). Those residues could be involved in the overall conformation of the active site or may have a direct role in the reaction by interacting with the substrate. The other three positively selected sites, N200, Q203 and R284 cluster on the surface interface between the molecules of the dimer. That interface has been shown in D-NCAase to form a hydrophobic pocket that is responsible for the tight dimer structure. It is known that the quaternary structures of nitrilases and cyanide hydratases can be quite different, ranging in size from monomers and dimers to oligomers containing 10, 14 or more subunits. Substrate binding has also been shown to play a role in the formation of active enzyme oligomers. The three interface residues may play a role in aspects of quaternary structure and substrate specificity associated with the proposed neofunctionalization after the cluster transition event.

## Conclusion

In this study, we combined genomic and biochemical analysis of a microbial enzyme family to understand evolutionary events that have shaped the genome organization and metabolism of organisms inhabiting various environments. It has long been known that bacterial genes often cluster based on linked functions. The gene location sometimes correlates with the order of the individual reactions in an enzymatic cascade or facilitate regulatory mechanisms of gene expression. Various models have been proposed to explain the formation, the evolutionary and physiological significance of operons and other gene clusters [23]. Comparative genomic studies have shown that recognition of clusters can assist in functional annotation of novel genes but clusters often they break apart with increasing taxonomic distance [49-53]. The Nit1C cluster that we described is remarkable in that it is highly conserved across several bacterial phyla and is present in organisms that inhabit extremely diverse environments. While limited rearrangements have occurred in Nit1C, the preservation of all seven genes suggests there is selective pressure for maintenance of the entire gene cluster regardless of the genomic dynamics in that neighborhood. The internal rearrangements of Nit1C correlate with high level taxa (cyanobacteria, beta and gamma proteobacteria).

There is no experimental evidence for an involvement of any of the Nit1C genes in a known metabolic transformation. Two of the cluster genes have no close homologs or predictable biochemical activities while the remaining genes, even though have a predictable type of biochemical activity, belong to classes of enzymes that are involved in a wide range of transformations. Predicting function for remote homologs in the absence of experimental data is still a major difficulty in genomics [54,55]. Having a defined cluster of genes such as Nit1C, likely to be functionally connected, sets the ground for future experimental genetic and biochemical investigation in search of its biological function.

Phylogenetically, the nitrilases from the Nit1C cluster appear strictly confined to a basal subset of subfamily 1 genes. More recent diversification of the genes in this subfamily has been accompanied by a change in the type of associated gene clusters and is paralleled by changes in biochemical properties of the nitrilases. While overall, subfamily 1 nitrilases are under strong purifying selection pressure, we detected a significant positive selection signal for the lineage following the transition event and identified several residues under such selection. This supports a hypothesis that a group of nitrilases diverged functionally from the Nit1C-type enzymes, became associated with other metabolic enzymes possibly as part of a novel pathway and advantageous mutations were fixed at specific sites under positive selection. Future studies of bacterial nitrilases and biochemical and genetic characterization of mutations at these residues are needed to better understand the determinants of substrate specificity and the functional differences between the nitrilase subfamilies.

Environmental microbial genomics has demonstrated its utility in studying large scale ecological processes [5,6,11], discovering valuable biocatalysts [15] and reassembling the genomic and metabolic blueprint of natural microbial communities thorough shotgun sequencing [7,8,10]. Vast amounts of sequence data could potentially be used to answer a wide range of questions, although there are open questions regarding experimental design, data analysis and breadth of biological significance [4,56,57]. A broad environmental sampling from worldwide geographical locations coupled with experimental biochemical validation and comparative genomic analysis allowed us to test metabolic and evolutionary hypotheses difficult to approach by using sequence data from only a few environments.

## Methods

### DNA sequences

The nitrilase sequences discovered from environmental DNA libraries are available from Genbank (AY487426-AY487562). Nitrilase sequences from sequenced bacterial genomes and their corresponding flanking genes were also obtained from GenBank, their names and accession numbers being indicated in the corresponding figures. For *Verrucomicrobium spinosum *DSM 4136, preliminary sequence data was obtained from the The Institute for Genome Research website [58] and for *Burkholderia fungorum *and *Rubrivivax gelatinosus *from the DOE Joint Genome Institute website [59].

### Enzymatic activity

The biochemical characterization data used in this study for the environmental nitrilases tested on the non physiological substrate hydroxyglutaronitrile has been published [9].

### Sequence analysis and annotation

For the analysis of the ORFs flanking the nitrilase genes in known bacterial genomes we used the sequence coordinates available in the corresponding GenBank files. For the environmental DNA clones containing nitrilase genes we identified and annotated the other open reading frames (ORFs) contiguous with the nitrilase in the genomic insert using standard approaches. The inserts varied in size from 1 to 7 kb and in most cases contained information to identify at least one or more ORFs in addition to the nitrilase gene. Annotation was derived based on available experimental or predicted function or biochemical activity using information associated with those genes in GenBank, PFAM, COG and KEGG databases.

### Phylogenetic reconstructions

Amino acid sequences were aligned in BioEdit [60] followed by manual refinement. Sequence alignments are provided [see Additional files 4, 5]. Phylogenetic trees were constructed in PROML (PHYLIP 3.6) [61] using maximum likelihood, JTT amino acid substitution matrix, five global rearrangements with randomized sequence input order and among-site rate variation modeled with an eight rate category discrete approximation to a gamma distribution. The model parameters were estimated using TREE-PUZZLE 5.1. [62]. Branch support was obtained by bootstrapping (100 replicates).

### Analysis for positive selection

A DNA sequence alignment for the nitrilase genes was obtained based on the protein alignment and used for phylogenetic reconstructions in PAUP* 4.0 [63] using maximum likelihood and is provided [see Additional file 6]. The model of sequence evolution (GTR+I+G) was selected using Modeltest v.3.06 [64]. To test specific branches for possible rate changes we used Hy-Phy [36]. The topologies for the DNA tree and the protein tree were identical.

The tree topology was used in the program codeml (PAML [65], to estimate dN/dS ratios based on maximum likelihood codon substitution models. Two categories of models were used, site specific [44] as well as branch-site models [46]. Statistical comparisons between the results from different nested models were done using likelihood ratio tests [66].

### Molecular modeling

A three-dimensional model for a clade 1 nitrilase (1A21) was obtained based on the structure of the homologous protein N-carbamoyl-D-amino acid amidohydrolase [48], using the Jackal software [67]. Analysis of the model and mapping of amino acid residues involved in catalysis or subject to positive selection was done in PyMol [68].

## Authors' contributions

MP participated in the design of the study, performed phylogenetic, comparative genomic and statistical analyses and drafted the manuscript. JE performed sequence analysis and functional annotation. TR participated in the design of the study, performed comparative genomic and gene function analyses. All authors contributed to the writing and approved the final manuscript.

## Supplementary Material

Additional file 1 Protein neighbor-joining tree for nitrilase genes from cultivated bacteria and from environmental samples. The environmental sequences are represented by GenBank accession numbers and gene names for those derived from Robertson *et al*, 2004. The Sargasso Sea sequences are shaded. Click here for file

Additional file 2 Maximum likelihood phylogenetic trees for genes that belong to the Nit1C clusters identified in known bacterial species, in the context of their respective protein families. Numbers represent bootstrap support (for major clades only). The Nit1C ORF sequences are shaded. Click here for file

Additional file 3 Maximum likelihood phylogenetic trees for two genes associated with nitrilases after the subfamily 1 cluster transition event, in the context of their respective larger protein families. The nitrilase associated genes are shaded. Numbers represent bootstrap support (for major clades only). Click here for file

Additional files 4 Alignment of nitrilase amino acid sequences from cultivated bacteria (used to generate the tree in Figure 1) Click here for file

Additional files 5 Alignment of nitrilase amino acid sequences used to generate the tree in Figure 3. Click here for file

Additional file 6 Alignment of DNA sequences of nitrilase genes used to test for positive selection and to generate the tree in Figure 4. Click here for file
