# Supplementary figures and images for: Evolution of a microbial nitrilase gene family: a comparative and environmental genomics study
Source: BMC Evol Biol. 2005 Aug 6;5:42. doi: 10.1186/1471-2148-5-42 (PMC1199592; doi:10.1186/1471-2148-5-42)

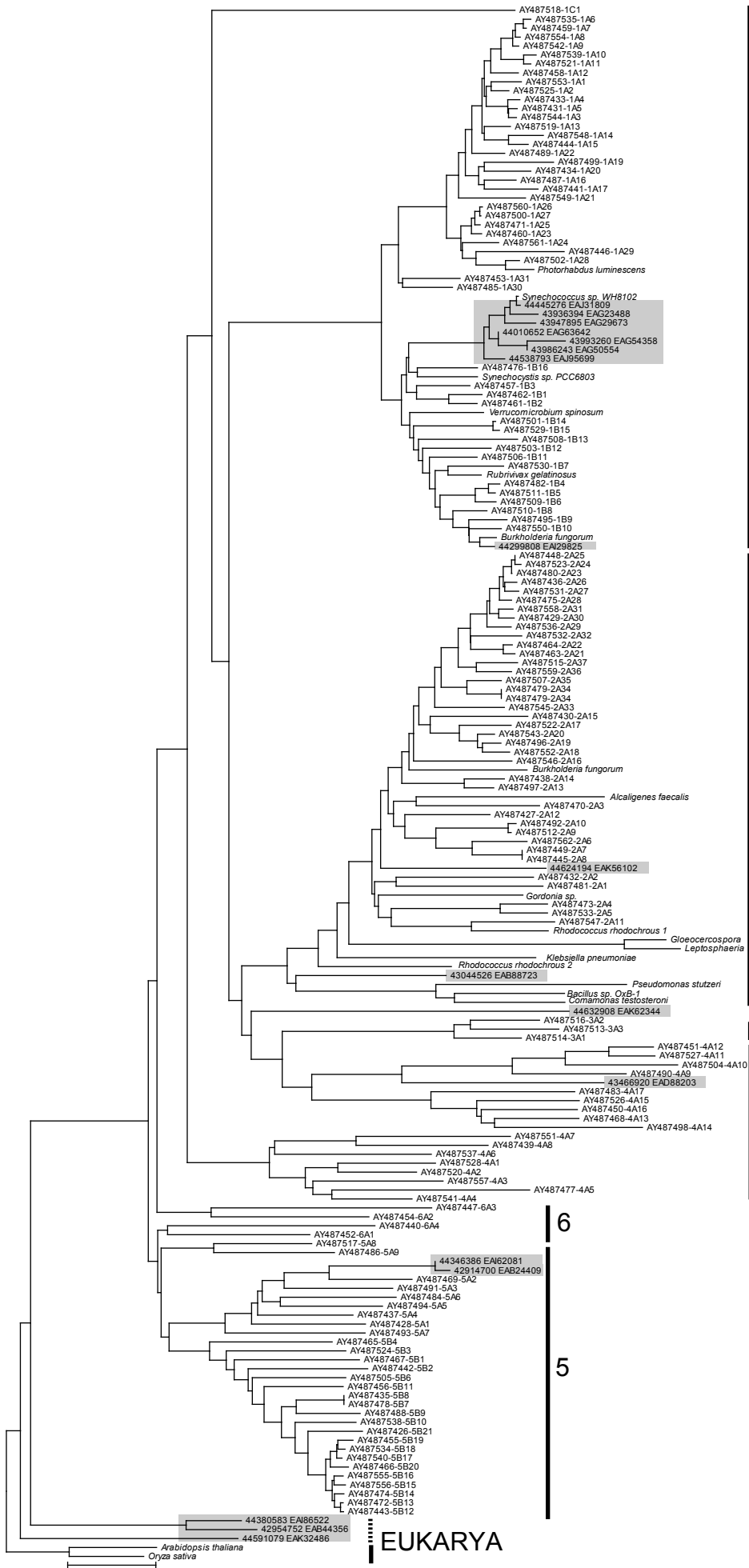

1

2

3

4

6

5

EUKARYA

0.2

Supplement: Additional file 1 — Protein neighbor-joining tree for nitrilase genes from cultivated bacteria and from environmental samples. The environmental sequences are represented by GenBank accession numbers and gene names for those derived from Robertson et al, 2004. The Sargasso Sea sequences are shaded. [file 1471-2148-5-42-S1.pdf]
