# Supplementary material for: Evolution of a microbial nitrilase gene family: a comparative and environmental genomics study
Source: BMC Evol Biol. 2005 Aug 6;5:42. doi: 10.1186/1471-2148-5-42 (PMC1199592; doi:10.1186/1471-2148-5-42)
Supplement: Additional file 2 — Maximum likelihood phylogenetic trees for genes that belong to the Nit1C clusters identified in known bacterial species, in the context of their respective protein families. Numbers represent bootstrap support (for major clades only). The Nit1C ORF sequences are shaded. [file 1471-2148-5-42-S2.pdf]

Nit1C ORF 3
COG2516: Biotin synthase-related enzyme
PF04055 Radical SAM superfamily

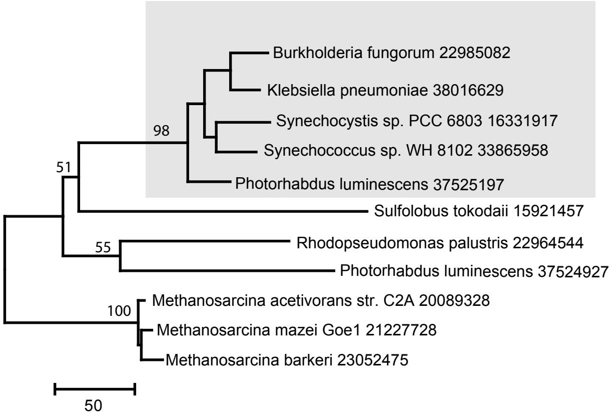

Ln Likelihood = -7127.8

Nit1C ORF 4
COG0454: Histone acetyltransferase HPA2 and related
PF00583 Acetyltransferase (GNAT) family

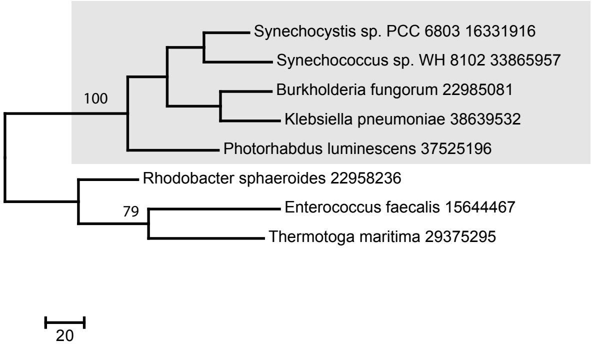

Ln Likelihood = -2200.884

Nit1C ORF 5
COG2144, PF00586 (AIRS)
AIR synthase , selenophosphate synthase

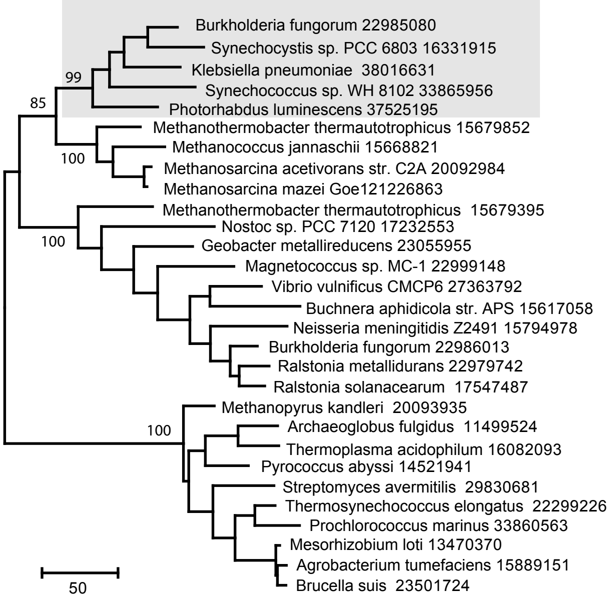

Ln Likelihood = -9914.1

Nit1C ORF 7
COG 2072, PF00070
pyridine nucleotide-disulphide oxidoreductase

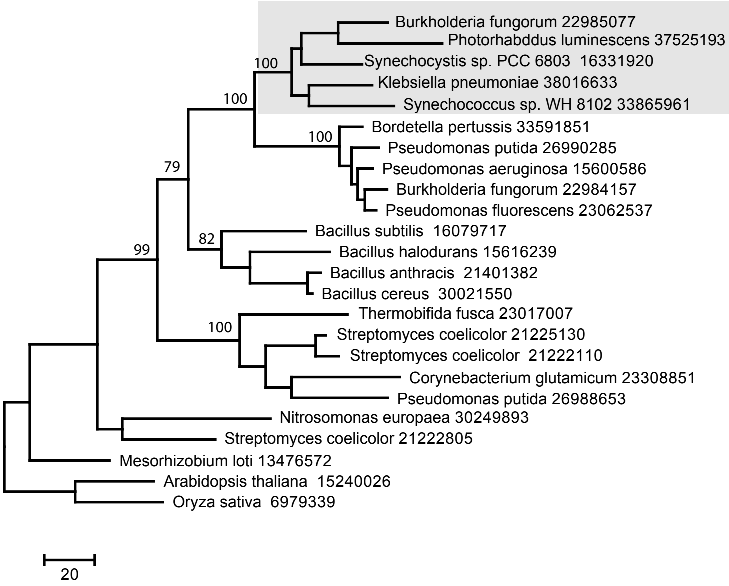

Ln Likelihood = -5294.59
