# Supplementary material for: Evolution of a microbial nitrilase gene family: a comparative and environmental genomics study
Source: BMC Evol Biol. 2005 Aug 6;5:42. doi: 10.1186/1471-2148-5-42 (PMC1199592; doi:10.1186/1471-2148-5-42)
Supplement: Additional file 3 — Maximum likelihood phylogenetic trees for two genes associated with nitrilases after the subfamily 1 cluster transition event, in the context of their respective larger protein families. The nitrilase associated genes are shaded. Numbers represent bootstrap support (for major clades only). [file 1471-2148-5-42-S3.pdf]

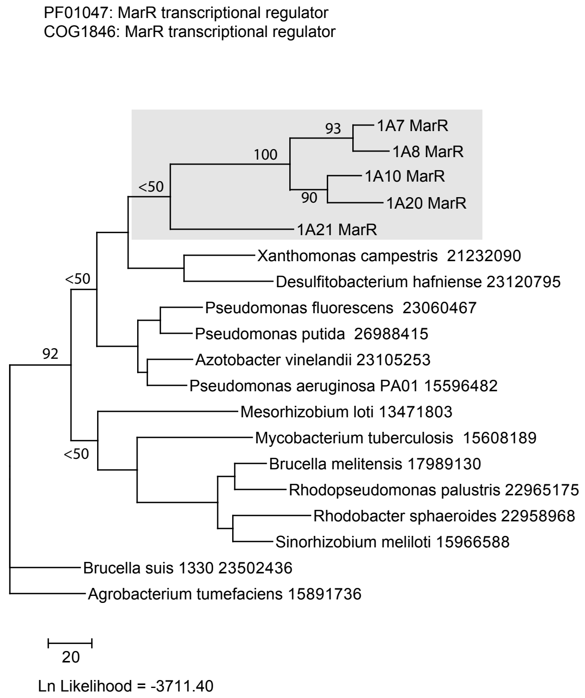
PF01047: MarR transcriptional regulator
COG1846: MarR transcriptional regulator
93
1A7 MarR
100
1A8 MarR
1A10 MarR
<50
90
1A20 MarR
1A21 MarR
Xanthomonas campestris 21232090
Desulfitobacterium hafniense 23120795
<50
Pseudomonas fluorescens 23060467
Pseudomonas putida 26988415
92
Azotobacter vinelandii 23105253
Pseudomonas aeruginosa PA01 15596482
Mesorhizobium loti 13471803
Mycobacterium tuberculosis 15608189
<50
Brucella melitensis 17989130
Rhodopseudomonas palustris 22965175
Rhodobacter sphaeroides 22958968
Sinorhizobium meliloti 15966588
Brucella suis 1330 23502436
Agrobacterium tumefaciens 15891736
20
Ln Likelihood = -3711.40

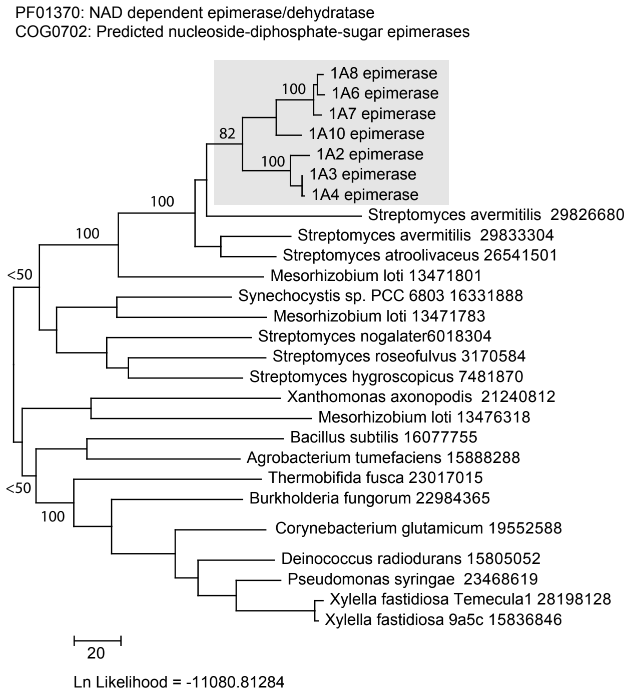
PF01370: NAD dependent epimerase/dehydratase
COG0702: Predicted nucleoside-diphosphate-sugar epimerases
1A8 epimerase
100
1A6 epimerase
1A7 epimerase
82
1A10 epimerase
100
1A2 epimerase
1A3 epimerase
1A4 epimerase
100
Streptomyces avermitilis 29826680
100
Streptomyces avermitilis 29833304
Streptomyces atroolivaceus 26541501
<50
Mesorhizobium loti 13471801
Synechocystis sp. PCC 6803 16331888
Mesorhizobium loti 13471783
Streptomyces nogalater6018304
Streptomyces roseofulvus 3170584
Streptomyces hygroscopicus 7481870
Xanthomonas axonopodis 21240812
Mesorhizobium loti 13476318
Bacillus subtilis 16077755
Agrobacterium tumefaciens 15888288
<50
Thermobifida fusca 23017015
Burkholderia fungorum 22984365
100
Corynebacterium glutamicum 19552588
Deinococcus radiodurans 15805052
Pseudomonas syringae 23468619
Xylella fastidiosa Temecula1 28198128
Xylella fastidiosa 9a5c 15836846
20
Ln Likelihood = -11080.81284
